# An uncommon presentation of eosinophilic granulomatosis with polyangiitis: a case report

**DOI:** 10.1186/1752-1947-8-190

**Published:** 2014-06-13

**Authors:** Giuseppe Taormina, Giuseppe Andolina, Maria Aurelia Banco, Edy Julia Costanza-Gaglio, Alice Bonura, Silvio Buscemi

**Affiliations:** 1 Internal Medicine and Hypertension Unit - Dipartimento Biomedico di Medicina Interna e Specialistica (DIBIMIS), P. Giaccone Policlinico of the University of Palermo, Via del Vespro 129, 90127 Palermo, Italy; 2 Cardiology Unit, P. Giaccone Policlinico of the University of Palermo, 90127 Palermo, Italy; 3 Section of Radiological Sciences, P. Giaccone Policlinico of the University of Palermo, 90127 Palermo, Italy

**Keywords:** Churg–Strauss syndrome, Corticosteroids, Eosinophilic granulomatosis with polyangiitis, Self-medication, Stroke

## Abstract

**Introduction:**

Eosinophilic granulomatosis with polyangiitis is a rare and potentially fatal disease if not readily diagnosed. Cerebral involvement is extremely rare and clinical presentation as hemorrhagic stroke is even rarer.

**Case presentation:**

A 58-year-old Caucasian man was admitted to our medical unit because of a computed tomography-diagnosed hemorrhagic stroke with right-sided hemiparesis and fever. A chest computed tomography scan also revealed multiple bilateral pulmonary infiltrates; coronary artery, and carotid and left vertebral artery calcifications were also observed. Empiric antimicrobial therapy with cephalosporins was promptly undertaken; low-molecular-weight heparin was introduced as prophylaxis for venous thromboembolism. Over the following days, magnetic resonance imaging scans showed a regression of the hemorrhagic framework, also revealing hypoxic areas consistent with acute ischemic lesions. With a computed tomography scan showing a worsening of his pulmonary framework, antimicrobial therapy was modified and corticosteroids were introduced. A new blood cell count revealed further increased leukocytosis (17.49×10^3^μL), characterized by a surprising rise of eosinophilic cells (32.8%). Angiography of the coronary arteries found diffuse dilatations with severe signs of endothelial damage. Such an unexpected framework induced a strong suspicion that the stroke was the expression of a systemic vasculitis, which had triggered his cerebral, coronary, and pulmonary frameworks. The search for antineutrophil cytoplasmic antibody was positive for perinuclear antineutrophil cytoplasmic antibody, and eosinophilic granulomatosis with polyangiitis was diagnosed. Explaining to the patient the rarity of his disease, and what the most typical presentations of eosinophilic granulomatosis with polyangiitis were, he revealed that before admission he had had scalp injuries, in the nuchal region, and had taken corticosteroids as self-medication, with subsequent disappearance of the lesions. Therefore, high-dose corticosteroid treatment was started, and at discharge he was in good clinical condition with a slight right-sided hyposthenia.

**Conclusions:**

A diagnosis of eosinophilic granulomatosis with polyangiitis is often difficult, but we are convinced that intake of corticosteroids on a self-prescribed basis may have obscured the clinical presentation. Therefore, this case also suggests how the growing phenomenon of self-medication can be harmful, and that a careful investigation of clinical history is still an act of paramount importance.

## Introduction

Churg–Strauss syndrome or, as recently renamed, eosinophilic granulomatosis with polyangiitis (EGPA) [1], is a rare and potentially fatal disease, with an annual incidence ranging between 0.5 and 6.8 per million, and a prevalence of 10.7 to 14 per million [2]. This condition is defined by the presence of eosinophil-rich and necrotizing granulomatous inflammation, often involving the respiratory tract, and necrotizing vasculitis primarily affecting the small to medium vessels. Asthma and eosinophilia are highly associated with EGPA, and nasal polyps are common. Antineutrophil cytoplasmic antibodies (ANCAs) are frequent, particularly when glomerulonephritis is present [3]. Neurological involvement as peripheral neuropathy is a common feature, whereas cerebral involvement is extremely rare.

We present a case of an adult male athlete admitted for hemorrhagic stroke whose final diagnosis was EGPA.

## Case presentation

**Figure 1:**
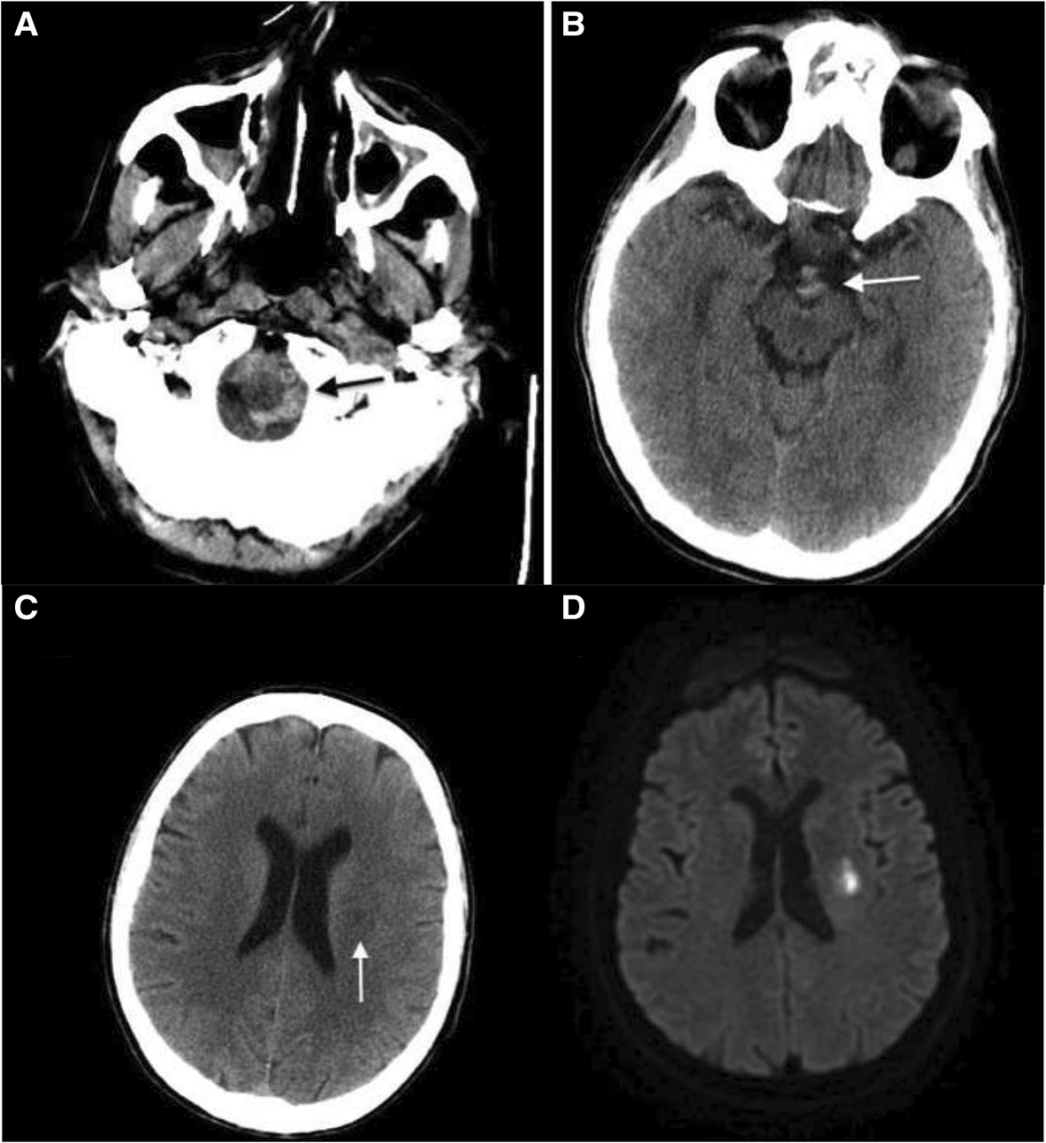
Initial non-contrast head computed tomography scan shows subarachnoid hemorrhage (arrows) (A, B); subsequent scan (C) shows ischemic lesion (arrow) that diffusion-weighted magnetic resonance imaging (D) confirms to be consistent with acute infarct.

**Figure 2:**
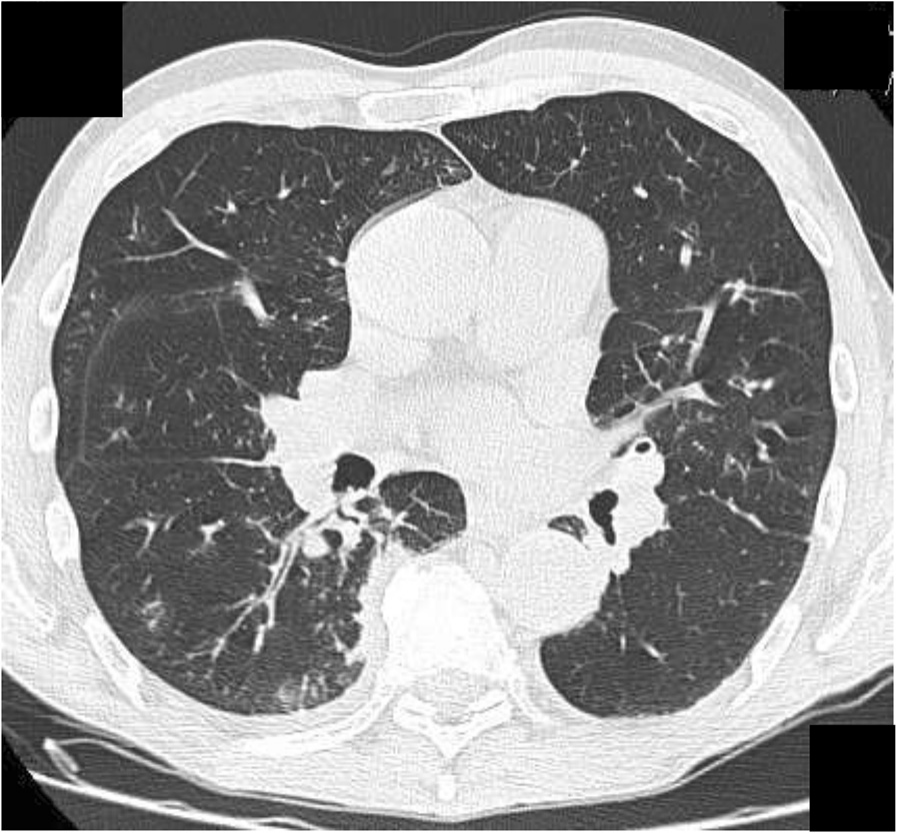
Non-contrast chest computed tomography scan shows multiple bilateral pulmonary infiltrates with ground-glass appearance.

**Figure 3:**
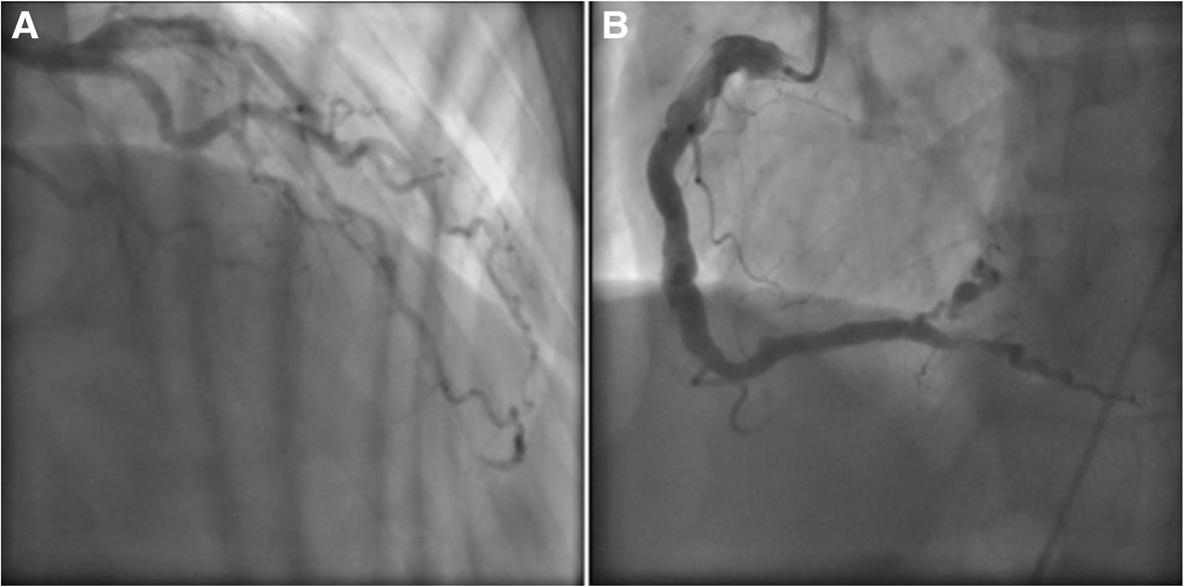
Angiography of left, circumflex (A) and right (B) coronary artery.

In mid-August 2013, a 58-year-old Caucasian man was admitted to our medical unit because of a hemorrhagic stroke with right-sided hemiparesis and fever. He was married and had two sons, had been employed at an electrical company, and had recently retired. He was an amateur marathon runner, covering about 5000km annually. The previous April he had taken part in a half-marathon, finishing fifth. His medical history was negative for cardiac symptoms or metabolic diseases. He had undergone a nasal polypectomy 7 years earlier and occasionally suffered from rhinitis and mild asthma, using low to middle doses of corticosteroids (prednisone 12.5 to 25mg per day) in periods of exacerbation on a self-prescribed basis. A neurological examination showed right-sided hemiparesis, particularly of his lower limb. He complained of tension headaches and an inability to maintain a standing posture. In the emergency area, a head computed tomography (CT) scan showed spontaneous hyperdensity of a hemorrhagic nature within his cisternal space adjacent to his medulla and the interpeduncular cistern; small hypoxic areas in his left corona radiata and insula were also reported (Figure 1A, B). A chest CT scan (Figure 2) also revealed multiple bilateral pulmonary infiltrates with ground-glass appearance localized in the apex of both upper lobes, in the posterior segment of the right upper lobe, and in the posterior basal segment of the right lower lobe. Surprisingly, calcifications in his coronary artery, and his carotid and left vertebral arteries, were also observed. On admission, laboratory tests revealed the presence of increased serum concentrations of inflammatory markers (C-reactive protein 7.85mg/dL, erythrocyte sedimentation rate 35mm/hour) and leukocytosis (13.8410^3^μL white blood cells, WBC) with neutrophilia (9.62×10^3^μL, 80.5%). Because of the pulmonary framework, empiric antimicrobial therapy with cephalosporins (ceftazidime 6g/day intravenous) was promptly undertaken. Given the immobilization, and as prophylaxis for venous thromboembolism, low-molecular-weight heparin (LMWH) was introduced. Over the following days, several head CT scans showed a regression of the hemorrhagic framework, and confirmed the hypoxic areas in his left corona radiata and insula consistent with acute ischemic lesion (Figure 1C). The latter finding was confirmed by head magnetic resonance imaging (MRI; Figure 1D) and magnetic resonance angiography, showing a hematic effusion within the lateral ventricles on a fluid attenuated inversion recovery sequence and, moreover, several punctiform ischemic lesions predominantly sided in the subcortical areas of his cerebral hemispheres, and another ischemic lesion in correspondence to the corona radiata and the insula. Intracranial vascular malformations and other abnormalities were excluded. Subsequently, a chest CT scan showed a worsening of the pulmonary framework. As a result, antimicrobial therapy was modified with the introduction of teicoplanin plus levofloxacin and corticosteroids (betamethasone 4mg/day intravenous). A new blood cell count revealed increased values of inflammatory markers with leukocytosis (17.49×10^3^μL WBC), characterized by a surprising rise in eosinophilic cells to 5.7×10^3^μL (32.8%). Furthermore, high levels of serum immunoglobulin E (3721mg/dL) were found. Because of the minute ischemic lesions revealed by head MRI, and to rule out a cardiogenic thromboembolism, on the 6th day ultrasonic cardiography was done, but was negative. The day after, angiography was performed in order to exclude supra-aortic vessels thrombosis and coronary disease because the previous chest CT scan had revealed the presence of coronary calcifications (the electrocardiogram was normal). The supra-aortic vessels were free of relevant lesions but, surprisingly, the coronary arteries had diffuse dilatations and stenosis with severe signs of endothelial damage. In particular, angiography demonstrated extensive and severe parietal alterations throughout the course of the left coronary artery, obstructed at the end of the second section, extensive and severe parietal alterations of the left circumflex artery with moderate distal stenosis. (Figure 3A) and right coronary artery critical stenosis with diffuse and severe alterations throughout its course (Figure 3B). Such an unexpected framework of endothelial damage induced a strong suspicion that the stroke was the expression of a systemic vasculitis, which had triggered the cerebral, coronary, and pulmonary frameworks. The further increase in circulating eosinophil cells (42.6%, 7.4×10^3^μL) led us to search for ANCA, which was positive for perinuclear-ANCA (p-ANCA; 1:200; reference value <1:20). A peripheral blood smear showed no presence of circulating blast cells.

**Figure 4:**
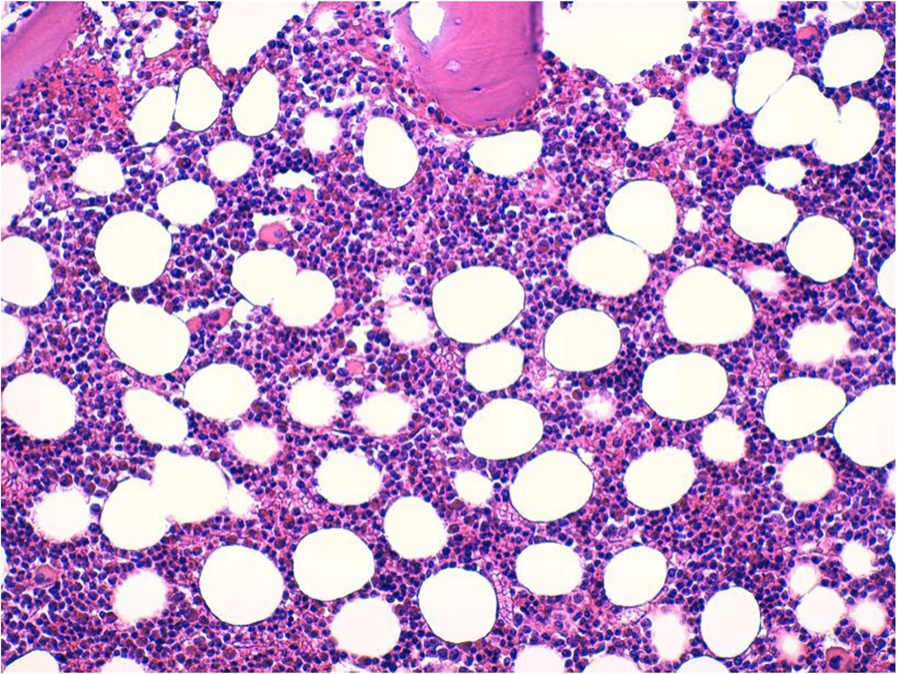
Bone marrow biopsy shows an increase of eosinophils with normal maturation (Giemsa, 200×).

A bone marrow aspiration was done to exclude a lymphoproliferative disorder, and revealed an increase in eosinophilic granulocytic series, with normal morphology in all stages of growth (Figure 4). A search for the *Abr/Bcl* gene was negative. A nasal mucus membrane biopsy showed no significant alterations. Urinary sediment analysis resulted in no relevant findings, nor did a renal artery ultrasonography examination or the indirect evaluation of intrarenal resistances (intrarenal resistance index and pulsatility index).

With reference to the American College of Rheumatology criteria [4] (Table 1), and because of the concomitance of pulmonary infiltrates, central nervous system involvement, blood eosinophilia, prior history of pulmonary asthma and nasal polyposis, and p-ANCA positivity, EGPA was diagnosed. Explaining to the patient the rarity of his disease, and what the most typical presentations of EGPA were, he revealed that because of the onset of scalp injuries in the nuchal region he had taken prednisone 12.5mg as self-medication for the 5 days preceding admission, with the disappearance of the lesions. Therefore, high-dose corticosteroid treatment was started (prednisone 1mg/kg per day), with a prompt regression of blood eosinophilia and respiratory symptoms, another factor supporting our diagnosis. He was discharged after 22 days of hospitalization, in good clinical condition, able to ambulate, and with a slight right-sided hyposthenia. As regards his follow-up, it was done on an out-patient basis at our department, with the aim of gradually reducing the corticosteroid dose, and tapering it to the maintenance dose. Two months after discharge, he was still in good clinical condition: the neurological impairment was greatly reduced and consisted only of a slight right-sided hyposthenia of his lower limb. There were no respiratory or cardiovascular symptoms, his WBC count was normal, and our advice was to remain under our observation for at least 2 years.

**Table 1:** **Churg–Strauss syndrome (The American College of Rheumatology 1990 diagnostic criteria **[4]**)**

Asthma	History of wheezing or diffuse high-pitched expiratory rhonchi
Eosinophilia	Eosinophilia >10% on differential white blood cell count
Mono- or polyneuropathy	Development of mononeuropathy, multiple mononeuropathies, or polyneuropathy (glove/stocking distribution) attributable to systemic vasculitis
Pulmonary infiltrates, non-fixed	Migratory or transitory pulmonary infiltrates (not including fixed infiltrates) attributable to vasculitis
Paranasal sinus abnormality	History of acute or chronic paranasal sinus pain or tenderness or radiographic opacification of the paranasal sinuses
Extravascular eosinophils	Extravascular areas

The presence of any four or more of the six criteria yields a sensitivity of 85% and a specificity of 99.7%.

## Discussion

EGPA, also known as Churg–Strauss Syndrome [1], is a small vessel ANCA-associated vasculitis typically characterized by the triad asthma, eosinophilia and vasculitis. Alternatively, EGPA is described as having three progressive phases, namely, a prodromal “allergic/atopic” phase of asthma and rhinosinusitis [5], an eosinophilic phase in which eosinophil-rich inflammatory tissue infiltrates develop, and a vasculitic phase the manifestations of which are similar to other ANCA-associated vasculitides, such as palpable purpura or mononeuritis multiplex. In a 32-year study of 96 patients with EGPA, asthma was the most common presentation, followed by mononeuritis, whereas stroke was an infrequent symptom [6]. Asthma in EGPA occurs in almost all cases (>95%), often severe and requiring steroids, anticipating by several years (7 to 8 years on average) the onset of the vasculitic phase. Upper airway involvement occurs in 70 to 90% of cases in the form of a rhinosinusitis, often in the presence of nasal polyposis. Pulmonary involvement (70 to 90%) consists of irregular, migratory and bilateral infiltrates with areas of ground-glass appearance. Extrapulmonary manifestations can include constitutional symptoms (50 to 90%), musculoskeletal (50%) or cutaneous disease (40 to 70%), peripheral nervous system (>50%), cardiac (30 to 50%) and gastrointestinal (30 to 50%) involvement. Many patients with EGPA do not have glomerulonephritis. Of interest, only 25% of patients with EGPA who have no renal disease are ANCA-positive, whereas 75% with any renal disease, and 100% with documented necrotizing glomerulonephritis, have ANCA positivity [3]. Significant ANCA serum concentrations are found in approximately 40% of patients with EGPA, and most of these patients are p-ANCA positive (anti-myeloperoxidase antibodies) [7].

We faced some difficulties in the approach to this clinical case. First, cerebral involvement is extremely rare. Indeed, the onset characterized by hemorrhagic stroke is absolutely atypical, and even more so when thrombotic findings are subsequently discovered. Recently, Go *et al*. [8] reported two clinical cases of EGPA with subarachnoid hemorrhage and intracerebral hemorrhage, respectively, and according to their review of the literature only 11 other cases of intracranial hemorrhage in association with EGPA had been reported. Second, the typical EGPA cutaneous manifestations were absent. Furthermore, there was no blood eosinophilia on admission. Despite the fact that a unique EGPA pathognomonic framework has yet to be described in the literature, we are convinced that intake of corticosteroids on a self-prescribed basis in the days immediately preceding the appearance of neurological symptoms may have obscured the clinical presentation. Therefore, this case suggests how the growing phenomenon of self-medication [9] can sometimes be harmful [10].

Certainly, the picture revealed by head MRI, with a hemorrhagic and multiple, small, left-side hypoxic areas, raised a strong suspicion that this was not a typical stroke. The progressive increase in eosinophils and pulmonary infiltrates, and the prior history of asthma and nasal polyposis, were important in arriving at a diagnosis. Of note secondary importance was the coronarographic response, which showed a diffuse and severe picture of endothelial damage, a further unmistakable sign of a systemic vasculitis. With this evidence, bone marrow aspiration seemed unnecessary. However, we decided to carry out this examination when the diagnosis was still uncertain and, given the complex spectrum of disorders related to serum eosinophilia [11], we had to exclude clonal disorders.

Univocal consensus on treatment of EGPA is still lacking, although, certainly, any therapeutic approach must be strictly related to the disease. Different scores are available to this purpose. In our case, the Birmingham Vasculitis Activity Score [12], the “five-factors score” of the French Vasculitis Study Group [13], and the European League Against Rheumatism guidelines [14], would have recommended inducing remission with immunosuppressants, such as cyclophosphamide, in addition to corticosteroids. However, given the prompt and favorable clinical response to high-dose corticosteroids in this specific case, we decided not to use immunosuppressants, unless first-line corticosteroid therapy failed. However, this is an already recognized clinical approach [15]. In any event, even when the diagnosis was still far from definitive, we gave the patient LMWH as thromboembolic prophylaxis. In addition, a low dosage of corticosteroids was promptly started because of the pulmonary picture. Given the definitive diagnosis, this therapeutic approach worked, although we did not know it would at the time. Minimum sequelae were detectable at discharge, and he was found in even better condition at subsequent follow-up visits. However, we remain convinced that the positive outcome of this case was fortunate because we began treatment without a definitive diagnosis, and that prompt treatment of the immediate clinical picture was paramount in resolving our patient’s condition.

## Conclusions

A diagnosis of EGPA is often difficult, but we are convinced that intake of corticosteroids on a self-prescribed basis may have mystified the clinical presentation. Therefore, this case also suggests how the growing phenomenon of self-medication can be harmful, and that a careful investigation of clinical history is still an act of paramount importance.

## Consent

Written informed consent was obtained from the patient for publication of this case report and accompanying images. A copy of the written consent is available for review by the Editor-in-Chief of this journal.

## Abbreviations

ANCA: antineutrophil cytoplasmic antibody; CT: computed tomography; EGPA: eosinophilic granulomatosis with polyangiitis; LMWH: low-molecular-weight heparin; MRI: magnetic resonance imaging; p-ANCA: perinuclear antineutrophil cytoplasmic antibody; WBC: white blood cells.

## Competing interests

The authors declare that they have no competing interests.

## Authors’ contributions

GT contributed to the clinical management of the patient, was responsible for acquisition of data, and edited the manuscript; GA performed the coronary angiography, and helped write the manuscript; MAB performed the radiology analysis and helped in writing the manuscript; EJC-G contributed to the review of the literature and the writing of the manuscript; AB contributed to the review of the literature and the writing of the manuscript; SB was responsible for the clinical management of the patient, and coordinated and edited the manuscript. All authors have read and approved the final manuscript.
